# Estrogen-dependent activation of TRX2 reverses oxidative stress and metabolic dysfunction associated with steatotic disease

**DOI:** 10.1038/s41419-025-07331-7

**Published:** 2025-01-31

**Authors:** Alfredo Smiriglia, Nicla Lorito, Marina Bacci, Angela Subbiani, Francesca Bonechi, Giuseppina Comito, Marta Anna Kowalik, Andrea Perra, Andrea Morandi

**Affiliations:** 1 Department of Experimental and Clinical Biomedical Sciences, University of Florence, 50134 Florence, Italy; 2 Department of Biomedical Sciences, University of Cagliari, 09042 Monserrato, Italy

**Keywords:** Cell biology, Molecular biology, Metabolic disorders

## Abstract

Metabolic dysfunction-associated steatotic liver disease (MASLD) encompasses a spectrum of hepatic disorders, ranging from simple steatosis to steatohepatitis, with the most severe outcomes including cirrhosis, liver failure, and hepatocellular carcinoma. Notably, MASLD prevalence is lower in premenopausal women than in men, suggesting a potential protective role of estrogens in mitigating disease onset and progression. In this study, we utilized preclinical in vitro models—immortalized cell lines and hepatocyte-like cells derived from human embryonic stem cells—exposed to clinically relevant steatotic-inducing agents. These exposures led to lipid droplet (LD) accumulation, increased reactive oxygen species (ROS) levels, and mitochondrial dysfunction, along with decreased expression of markers associated with hepatocyte functionality and differentiation. Estrogen treatment in steatotic-induced liver cells resulted in reduced ROS levels and LD content while preserving mitochondrial integrity, mediated by the upregulation of mitochondrial thioredoxin 2 (TRX2), an antioxidant system regulated by the estrogen receptor. Furthermore, disruption of TRX2, either pharmacologically using auranofin or through genetic interference, was sufficient to counteract the protective effects of estrogens, highlighting a potential mechanism through which estrogens may prevent or slow MASLD progression.

## Introduction

Metabolic dysfunction-associated steatotic liver disease (MASLD), formerly referred to as non-alcoholic fatty liver disease (NAFLD), encompasses a wide range of hepatic pathologies and functional impairments associated with at least one cardiometabolic criterion [1]. Key hepatic alterations in MASLD include steatosis, chronic inflammation (metabolic dysfunction-associated steatohepatitis, MASH), fibrosis, and cirrhosis. Notably, cirrhosis poses a significant risk for neoplastic transformation of hepatocytes, leading to hepatocellular carcinoma (HCC). Hepatic steatosis, defined by at least 5% lipid accumulation within liver tissue, is considered reversible; however, prolonged and severe MASH is recognized as a progressive disease [2].

Numerous molecular pathways contribute to MASLD onset, driving the phenotypic traits observed in hepatic cells undergoing steatosis [3–5]. This condition is typically characterized by excessive intracellular triglyceride accumulation independent of alcohol consumption, accompanied by inflammation and oxidative stress—both key factors that promote steatohepatitis [6].

Globally, MASLD affects approximately 25% of the population, with significantly lower incidence in premenopausal women compared to men and postmenopausal women [7], despite a higher prevalence of obesity among women—a primary risk factor for MASLD [8]. This disparity suggests a potential protective effect of estrogens in preventing or delaying MASLD initiation and progression. Mounting evidence supports the benefits of estrogen replacement therapy in mitigating lipid accumulation and peroxidation [9], reducing lipotoxicity-induced oxidative stress in hepatic mitochondria, and alleviating inflammation [10–12]. Studies in ovariectomized mice have demonstrated that supplementation with 17β-estradiol (E2) [13], the predominant circulating form of female hormones, inhibits steatogenesis [14]. However, the precise molecular mechanisms underlying the protective effects of estrogens in MASLD onset and progression remain insufficiently understood.

Evidence suggests that estrogens primarily prevent oxidative damage through estrogen receptor alpha (ER), which promotes the expression and activation of a range of antioxidant enzymes. Specifically, nuclear factor erythroid 2-related factor 2 (NRF2) and thioredoxin 2 (*TXN2*, which encodes the TRX2 protein) are ER-dependent genes involved in cellular defense against reactive oxygen species (ROS). TRX2 directly neutralizes ROS, while NRF2 regulates transcriptional activation of downstream scavenging mechanisms [15, 16]. These processes collectively exert anti-steatotic effects, alleviating inflammation associated with MASLD [17, 18].

In this study, we investigated the protective role of estrogens in counteracting oxidative stress through activation of the antioxidant mitochondrial TRX2 pathway, using preclinical in vitro models exposed to clinically relevant steatotic-inducing agents.

## Materials and methods

### Cell lines and general culture conditions

Male and female human embryonic stem cell (hESC) lines, WA01 and WA09 respectively, were purchased from WiCell Research Institute (504 S Rosa Rd #101, Madison, WI 53719, USA) and maintained at 37 °C/5% CO_2_ on 5 μg/mL laminin 521 (LMN-521, STEMCELL Technologies #LN521-05) pre-coated plates with mTeSR1 PLUS serum-free medium (STEMCELL Technologies #100-0276).

AML12 and HepG2 cell lines were purchased from American Type Culture Collection (ATCC): AML12 cells are hepatocytes isolated from the normal liver of a male mouse and grow in DMEM:F12 medium (1:1, ThermoFisher #11330032) supplemented with 10% fetal bovine serum (FBS), 10 µg/mL insulin (I), 5.5 µg/mL transferrin (T), 5 ng/mL selenium (S) (ITS, ThermoFisher #41400045), and 40 ng/mL dexamethasone (ThermoFisher #A13449); HepG2 is a human hepatoblastoma cell line of a 15-year-old male maintained in DMEM high glucose medium (ThermoFisher #11965092) supplemented with 10% FBS, 2 mM glutamax (ThermoFisher #35050061), and 1% penicillin/streptomycin (ThermoFisher #15140).

### Compounds

Auranofin (AU, Sigma-Aldrich #A6733) and 17β-Estradiol (E2, Sigma-Aldrich #E2758) were dissolved in DMSO and ethanol, respectively. The ATGL inhibitor (Atglistatin, ATGLi, N’-[4’-(dimethylamino)[1,1’-biphenyl]-3-yl]-N,N-dimethyl-urea, MedChemExpress #HY-15859) was dissolved in DMSO. All the compounds were used at the specified concentrations, as described in the figure legends.

### Differentiation from hESC to hepatocyte-like cells (HLC)

At the time of differentiation into HLC, hESC were plated as a single cell at a concentration of 400,000 cells per well onto the LMN-521 coating and 10 µM of Rho-associated kinase inhibitor Y27632 (ROCKi, Stem Cell Technologies #72302) were added to the medium. This inhibitor was used to improve the adhesion and survival of stem cells in single-cell suspension. After 24 h, when hESC reached a 40% confluence, we initiated the differentiation process in HLC, as accurately detailed in [19]. Briefly, WA01 and WA09 cells were initially differentiated in endodermal cells by culturing them in RPMI 1640 medium (Gibco #11875-093) containing B-27 Supplement, (Gibco #12587-010), 100 ng/mL Activin A (PeproTech #120-14E), and 50 ng/mL Wnt3A (Peprotech #315-20-10uG). Subsequently, the hepatoblast phenotype was obtained by differentiating endodermal cells in Knockout DMEM (Gibco, #10829), containing 20% Knockout Serum Replacement (Gibco #10828), 1% non-essential amino acids (NEAA, Gibco #11140), 0.1 mM 2-mercaptoethanol (Gibco #31350). Finally, hepatoblast cells were differentiated into HLC by culturing them, until day 17, in HepatoZYME medium (Gibco, #17705) containing 10 ng/mL hepatocyte growth factor (Peprotech, #100-39), 20 ng/mL oncostatin M (Peprotech, #300-10), and 10 µM hydrocortisone 21-hemisuccinate (Sigma-Aldrich, #H4881). At day 18, HLC were used for the assays described in this manuscript.

### Induction of steatotic and MASH phenotype

Hepatocyte steatosis was induced by administrating a mix of 10 mM sodium-L-lactate (L, Sigma-Aldrich #7022), 1 mM sodium pyruvate (P, Sigma-Aldrich P2256), and 2 mM octanoic acid (O, Sigma-Aldrich C2875), namely LPO, for 48 h to HLC, AML12, and HepG2 cells. MASH conditions are mimicked as previously reported [20]. Briefly, AML12 and HepG2 cells are exposed for 24 h to 65 μM sodium oleate (Sigma-Aldrich #O7501) and 45 μM palmitic acid (Sigma-Aldrich #P0500), 100 nM insulin (MedChemExpress #HY-P1156), 4.5 mg/mL glucose (Sigma-Aldrich #G8644) and inflammatory cytokines: 50 ng/mL tumor necrosis factor α (TNFα, Peprotech #HZ-1014), 25 ng/mL interleukin 1β (IL1β, Peprotech #HZ-1164) and 8 ng/mL transforming growth factor β (TGFβ, Peprotech #HZ-1011).

### siRNA transfection

AML12 and HepG2 cells were seeded into 6-well plates (25 × 10^4^ per well) to achieve 70% confluence the following day, when cells were transfected with either 50 nmol/L siRNA targeting TRX2 (human and mouse siTRX2 SMARTpool, GE Healthcare Dharmacon) or a negative control (non-targeting small interfering RNA, siCTR, GE Healthcare Dharmacon) using Lipofectamine RNAiMAX Reagent (ThermoFisher Scientific #13778-150) and Opti-MEM (GIBCO #31985062), accordingly to manufacturer’s instructions. The analyses were performed 72 h after transfection as described in figure legends.

### Western blotting analysis

Cell lines were washed with PBS and lysed in ice using Laemmli Sample Buffer 1X (Biorad #161-0737) supplemented with protease and phosphatase inhibitors (Sigma-Aldrich #P8340 and #P0044, respectively), and protein concentrations were measured by BCA (Sigma-Aldrich #1003290033) method. Then, 30–35 μg of cell lysate were loaded in precast SDS-PAGE (sodium dodecyl sulfate–polyacrylamide gel electrophoresis) gels (Biorad #456-8096) and then transferred onto nitrocellulose membrane by Trans-Blot Turbo Transfer Pack (Biorad #170-4157). The immunoblots were incubated in non-fat dry milk 5%, tween-20 0.05% in PBS at room temperature for 1 h, and then probed with primary and appropriate secondary antibodies. The following antibodies were used: HNF4α (Cell Signaling Technology #311F12), Albumin (Santa Cruz Biotechnology #sc-271605-F10), E-cadherin (Cell Signaling Technology #3195), TRX2 (Santa Cruz Biotechnology #sc-133201-F10), ER (Abcam # ab16660), ACTB (Cell Signaling Technology #4970), Hsp90 (Santa Cruz Biotechnology #sc-69703), and Histone H3 (Cell Signaling Technology #4499). Full and uncropped blots are provided in Supplementary Material.

### RNA extraction and quantitative Real-time PCR (qRT-PCR) analysis

Total RNA was extracted using RNeasy plus kit (QIAGEN #74134), quantified using Nanodrop 1000 (ThermoFisher Scientific), and 500 ng were reverse transcribed using the iScript gDNA Clear cDNA Synthesis Kit (Biorad #172-5035). qRT-PCR was performed using the CFX96 Touch Real-Time PCR Detection System (Biorad) using TaqMan Universal PCR Master Mix (ThermoFisher Scientific #4305719). The probes used in the work are: Albumin (Hs00609411_m1), HNF4α (Hs00230853_m1), TXN2 (Hs00429399_g1), PLIN2 (Hs00605340_m1 and Mm00475794_m1), GDF15 (Hs00171132_m1), SLC34A2 (Hs00197519_m1), NRF2 (Hs00975961_g1), NQO1 (Hs01045993_g1). Data were normalized on TBP (Hs00427620_m1) and/or ACTB (Mm02619580_g1). The relative quantity was determined using ^∆^Ct and ^∆∆^Ct by the CFX Maestro software (BioRad).

### Confocal image acquisition and analysis

Cells were plated at a concentration of 10,000 cells per well in a Nunc™Lab-Tek™Chamber Slide System coverglass (ThermoFisher Scientific #155383). Once steatosis had been induced, cells were stained at 37 °C for (1) 15 min with BODIPY^493/503^ (ThermoFisher Scientific #D3922) to reveal LD content, (2) 30 min with MitoSOX (ThermoFisher Scientific #M36008) and CellROX (ThermoFisher Scientific #C10444) to reveal mitochondrial and total ROS respectively, (3) 20 min with TMRE (tetramethylrhodamine, ethyl ester, ThermoFisher Scientific #T669) a cell-permeable, cationic dye that selectively accumulates within mitochondria due to their elevated membrane potential and negative charge, (4) 20 min with MitoTracker Green (ThermoFisher Scientific #M7514) used to assess mitochondrial mass. When the incubation with the probes was completed, cells were fixed using a 4% formaldehyde solution for 10 min. Subsequently, the formaldehyde was removed, and three washes were performed with PBS. To stain the nuclei, cells were then incubated for 10 min with the DAPI dye (excitation, ex: 358 nm; emission, em: 461 nm) diluted 1:1000 in PBS and subsequently washed an additional 3 times in PBS. Finally, images were acquired using a TCS SP8 microscope (Leica Microsystems) with LAS-AF image acquisition software. The quantification of intracellular lipids was performed using CellProfiler software from three representative 63x images from 3 independent experiments. ImageJ v.1.54k was used to determine the LD average size for cells that were stained using BODIPY^493/503^, the average mitochondrial aspect ratio (major axis length ÷ minor axis length), and the form factor for cells that were stained using TMRE [21]. Three representative cells per field of view were chosen from 3 technical replicates of at least 3 independent biological experiments. The aspect ratio serves as an indicator of mitochondrial elongation, while the form factor also accounts for the perimeter, making it more sensitive to curvature and the irregular shapes of filamentous mitochondria. Higher values for both the form factor and aspect ratio indicate increased mitochondrial branching and complexity.

### ROS analysis

Cell lines (7 × 10^4^ cells/well) were seeded into 12-well plates. Once steatosis was induced, the cells were incubated with the CellROX (ex: 485 nm; em: 520 nm) and MitoSOX (ex: 500 nm; em: 610 nm) probes in the dark for 30 min. Then, cells were lysed with RIPA buffer and fluorescence was measured on a Synergy H1 Hybrid Multi-Mode Reader (Biotek) plate reader.

### In silico analysis for TXN2 expression

Transcriptomic data are available at the National Center for Biotechnology Information GEO repository (accession number GSE163211). The GSE163211 dataset was analyzed with GEO2R and R-program. The data used represent the expression values of TXN2. The statistical test performed was the Wilcox test. Statistical significance was defined as *p* < 0.05.

### Statistical analysis

Statistics were performed using Prism 10 (GraphPad Software). Unless stated otherwise, all numerical data are expressed as the mean ± standard error of the mean (SEM). All experiments were conducted at least 3 times independently, with one or more technical replicates for each experimental condition tested. Each dot represents a replicate. The normality of data distribution was determined by Shapiro–Wilk or D’Agostino-Pearson omnibus (K2) test. Means of two sample groups were compared by an unpaired two-tailed Student’s *t*-test for normally distributed data, whereas Mann–Whitney nonparametric test was used for non-normally distributed data. For comparisons among multiple groups, one-way analysis of variance (ANOVA) test for normally distributed data or Kruskal–Wallis test for non-normally distributed data followed by Tukey or Dunnett’s post hoc analysis were performed. Statistical significance was defined as: *p < 0.05; **p < 0.01; ***p < 0.001; ****p < 0.0001; when differences were not statistically significant, or the comparison was not biologically relevant, no indication was reported in the figures. The treatment groups were randomized, but blinding was not implemented during the experimental processes. No sample was excluded from the study.

## Results

### Phenotypic and functional characterization of HLC derived from hESC

**Fig. 1:**
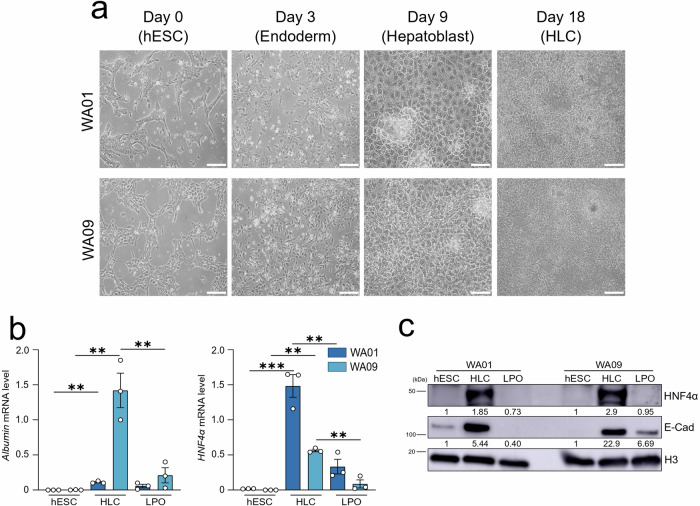
Phenotypic and functional characterization of HLC derived from hESC. **a** Representative bright field images of the differentiation phases that lead WA01 and WA09 hESC to differentiate into HLC. Scale bar, 40 µm. **b** hESC, HLC and LPO-treated HLC were subjected to qRT-PCR analysis. The relative quantity is shown using ^∆^Ct. Data represent means ± SEM. One-way ANOVA, Tukey-corrected. **p < 0.01; ***p < 0.001. Each dot represents a biological replicate, *n* = 3. **c** Total protein lysates from WA01 and WA09 hESC, derived HLC and LPO-treated HLC were subjected to western blot analysis with the antibodies indicated. The Histone H3 (H3) is used as a protein loading control normalizer.

Male WA01 and female WA09 hESC were differentiated into HLC using an established 18-day protocol [19]. The differentiation of hESC into HLC was confirmed by the acquisition of a classic cobblestone-like hepatic morphology (Fig. 1a), alongside the expression of established hepatic markers, including increased mRNA levels of albumin (Fig. 1b) and mRNA and protein levels of hepatocyte nuclear factor-4-alpha (HNF4α) in HLC compared to undifferentiated hESC (Fig. 1b, c). Additionally, we observed increased protein expression of E-Cadherin (E-Cad), a key molecule for the assembly and functionality of adherent junctions (Fig. 1c), which is a characteristic feature of differentiated hepatocytes. Thus, by differentiating sex-matched hESC into HLC, we established a relevant hepatocyte model suitable for studying the onset and progression of MASLD and assessing the impact of estrogen administration.

### Lactate, pyruvate, and octanoic acid-induced steatosis in HLC and immortalized cells

To comprehensively model steatosis in vitro, we utilized WA01 and WA09 hESC-HLC, alongside two well-established immortalized liver cell lines: AML12, hepatocytes isolated from normal murine liver tissue [22], and HepG2, a hepatoblastoma-derived cell line widely employed in liver cancer research [23].

The four cell models were treated with a compound cocktail containing sodium L-lactate (L), sodium pyruvate (P), and octanoic acid (O)—hereafter referred to as LPO—for 48 h to induce steatosis [24]. Following LPO treatment, both hESC-derived HLC models exhibited significant reductions in mRNA and/or protein expression levels of albumin, HNF4α, and E-Cad (Fig. 1b, c), indicating hepatocyte dysfunction.

**Fig. 2:**
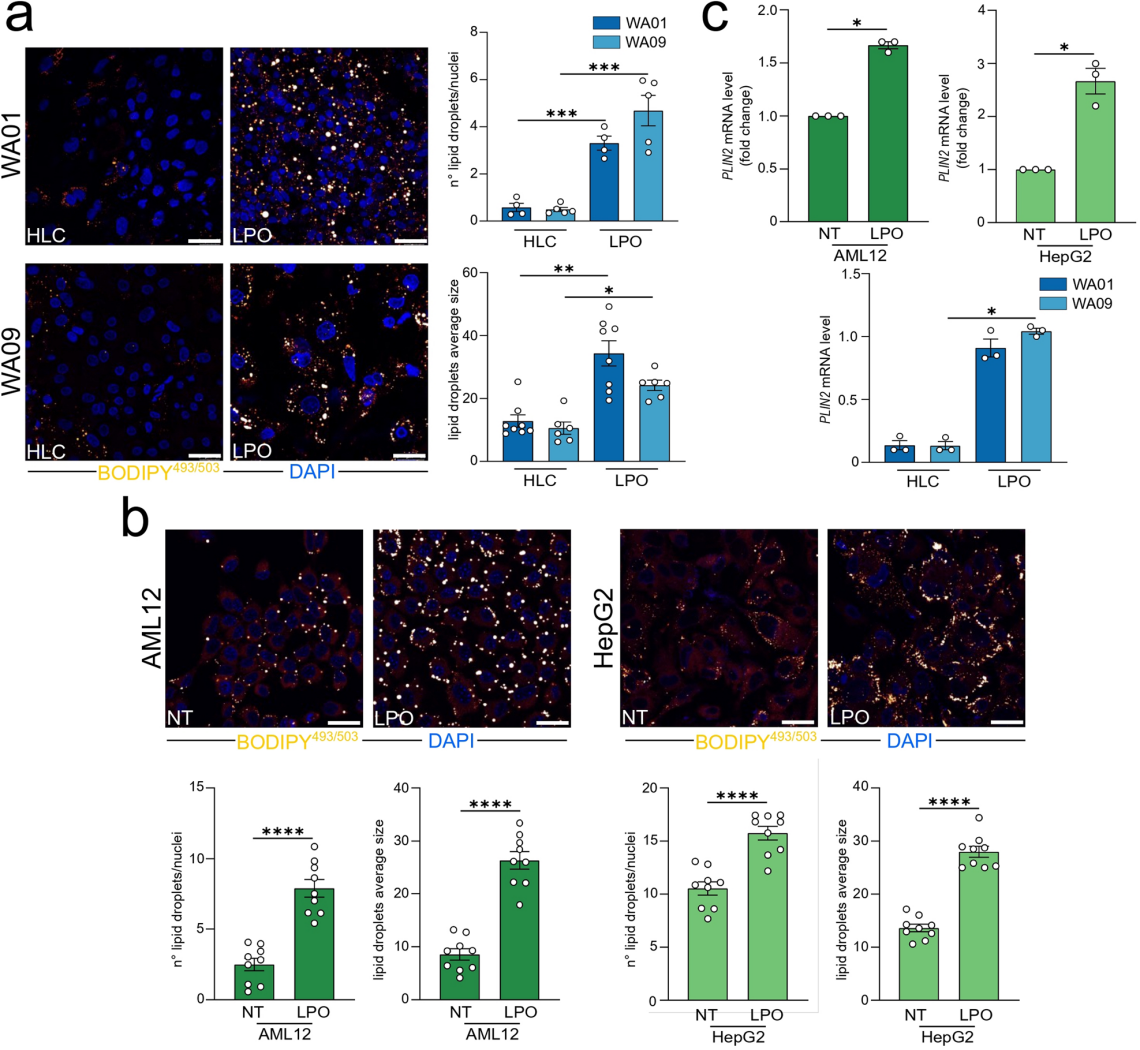
Lactate, pyruvate, and octanoic acid-induced steatosis in HLC and immortalized cells. Untreated and LPO-exposed **a** WA01 and WA09 HLC, **b** AML12, and HepG2 cells were subjected to confocal analysis as indicated in the figure. Representative confocal images of BODIPY^493/503^ stained cells are shown (orange/yellow: LD; blue: DAPI, nuclei. Scale bar, 25 µm). Quantification of LD number/cell and their average size (arbitrary unit) were reported. The confocal analysis of LPO-treated AML12 and HepG2 cells has been used as reference in Fig. 5b. **c** Untreated and LPO-exposed WA01, WA09, AML12, and HepG2 cells were subjected to qRT-PCR analysis. The relative quantity normalized on untreated is shown using ^∆∆^Ct for AML12 and HepG2 cells, whereas the relative quantity is shown using ^∆^Ct for WA01 and WA09 cells. Data represent means ± SEM. (**a**—n° lipid droplets; **b**; **c**—AML12 and HepG2) Student’s *t*-test, (**a**—average size) Mann–Whitney, (**c**—HLC) Kruskal–Wallis, Dunnett-corrected, *p < 0.05, ***p < 0.001, ****p < 0.0001. Each dot represents a biological replicate, *n* ≥ 3.

Steatosis is marked by the intracellular accumulation of lipids within hepatocyte cytoplasm in the form of lipid droplets (LD), which are dynamic and multifunctional organelles involved in energy metabolism, signaling, and inflammatory mediator production [25]. To determine if LPO treatment could similarly induce LD accumulation, we conducted confocal analysis using the fluorescent neutral lipid dye BODIPY^493/503^, which specifically stains LD. Confocal imaging and subsequent quantification revealed that LPO-treated WA01 and WA09 cells exhibited a significantly enhanced LD content, alongside an increase in average LD size (Fig. 2a). Similarly, LPO treatment substantially increased both the number and average size of LD in the AML12 and HepG2 cell lines (Fig. 2b). Concurrently, all LPO-exposed cell models displayed significantly elevated levels of PLIN2 (Fig. 2c), a perilipin family protein linked to LD accumulation [26, 27].

### LPO treatment increased oxidative stress in HLC and immortalized cells

**Fig. 3:**
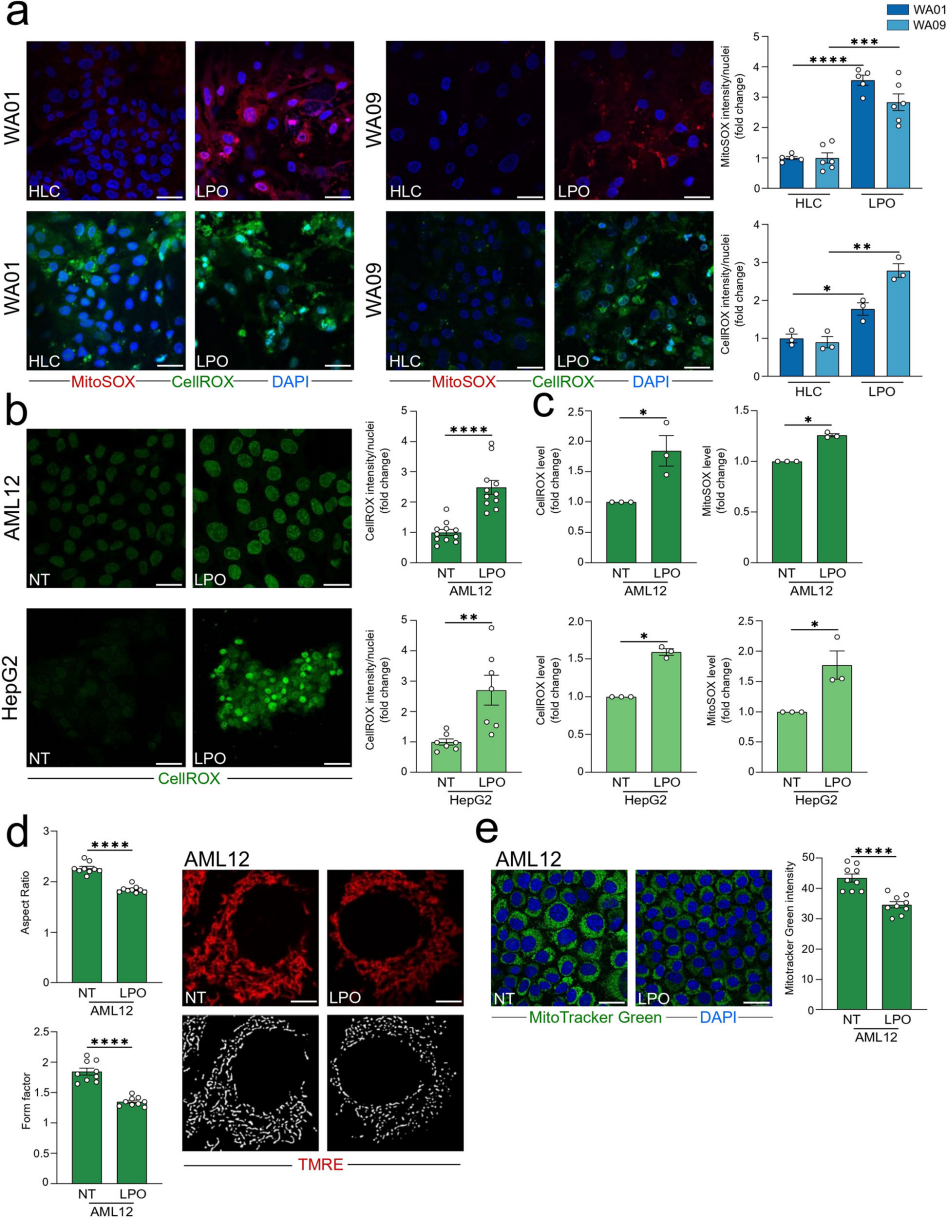
LPO treatment increased oxidative stress in HLC and immortalized cells. **a** Untreated and LPO-exposed (48 h) WA01 and WA09 cells were subjected to confocal analysis and subsequent quantification. Representative images of MitoSOX and CellROX-stained cells are shown (Red: MitoSOX; Green: CellROX; blue: DAPI, nuclei. Scale bar, 25 µm). **b**, **c** AML12 and HepG2 cells were treated with LPO for 48 h and subjected to confocal and fluorescent analyses. **b** Representative pictures of CellROX-stained cells are shown (Green: CellROX. Scale bar, 25 µm), *n* ≥ 3. Quantification of CellROX is reported. **c** ROS levels were measured using CellROX and MitoSOX fluorescent probes in AML12 and HepG2 cells treated with LPO (48 h). Untreated cells were used as comparator. **d** LPO-exposed AML12 cells was subjected to TMRE staining and subsequent quantification of mitochondrial morphology parameters: aspect ratio and form factor (Red: TMRE. Scale bar, 25 µm). The confocal analysis of LPO-treated AML12 cells has been used as reference in Fig. 4h. **e** Representative pictures of MitoTracker Green-stained AML12 cells are shown (Green: MitoTracker Green; blue: DAPI, nuclei. Scale bar, 25 µm). Quantification of MitoTracker Green is reported. The confocal analysis of LPO-treated AML12 cells has been used as reference in Fig. 4i. Data represent means ± SEM. **a**, **b**; **d**; **e** Student’s *t*-test, **c** Mann–Whitney, *p < 0.05, **p < 0.01; ***p < 0.001; ****p < 0.0001. **a**–**c** Each dot represents a biological replicate, **d**, **e** three biological replicates in technical triplicate.

In addition to LD accumulation, oxidative stress represents a key factor in the onset and progression of MASLD [28]. To assess this, we measured mitochondrial and total ROS levels using MitoSOX and CellROX fluorescent probes, respectively, which revealed elevated ROS levels in LPO-treated HLC compared to untreated (i.e., healthy) (Fig. 3a). Similarly, both AML12 and HepG2 cell lines exhibited increased total and mitochondrial ROS levels following LPO administration, as detected through confocal (Fig. 3b) and fluorometric (Fig. 3c) analyses. The increase in ROS levels upon LPO administration was accompanied by a reduction (1) in the mitochondrial morphological indicators, form factor and aspect ratio (Fig. 3d), indicative of a functional impairment of mitochondria consistent with previous reports [29], and (2) in mitochondrial mass (Fig. 3e). To validate these findings, we used a clinically relevant MASH-mimicking cocktail [20] containing sodium oleate, palmitic acid, insulin, glucose, and inflammatory cytokines (TNF-α, IL-1β, TGF-β) to induce MASH in AML12 and HepG2 cells. This cocktail effectively induced lipid accumulation and elevated both total and mitochondrial ROS in AML12 and HepG2 cells (Fig. S1a, b).

### Estrogens reduced oxidative stress, altered mitochondrial morphology, and LD accumulation in LPO-treated hepatocytes

**Fig. 4:**
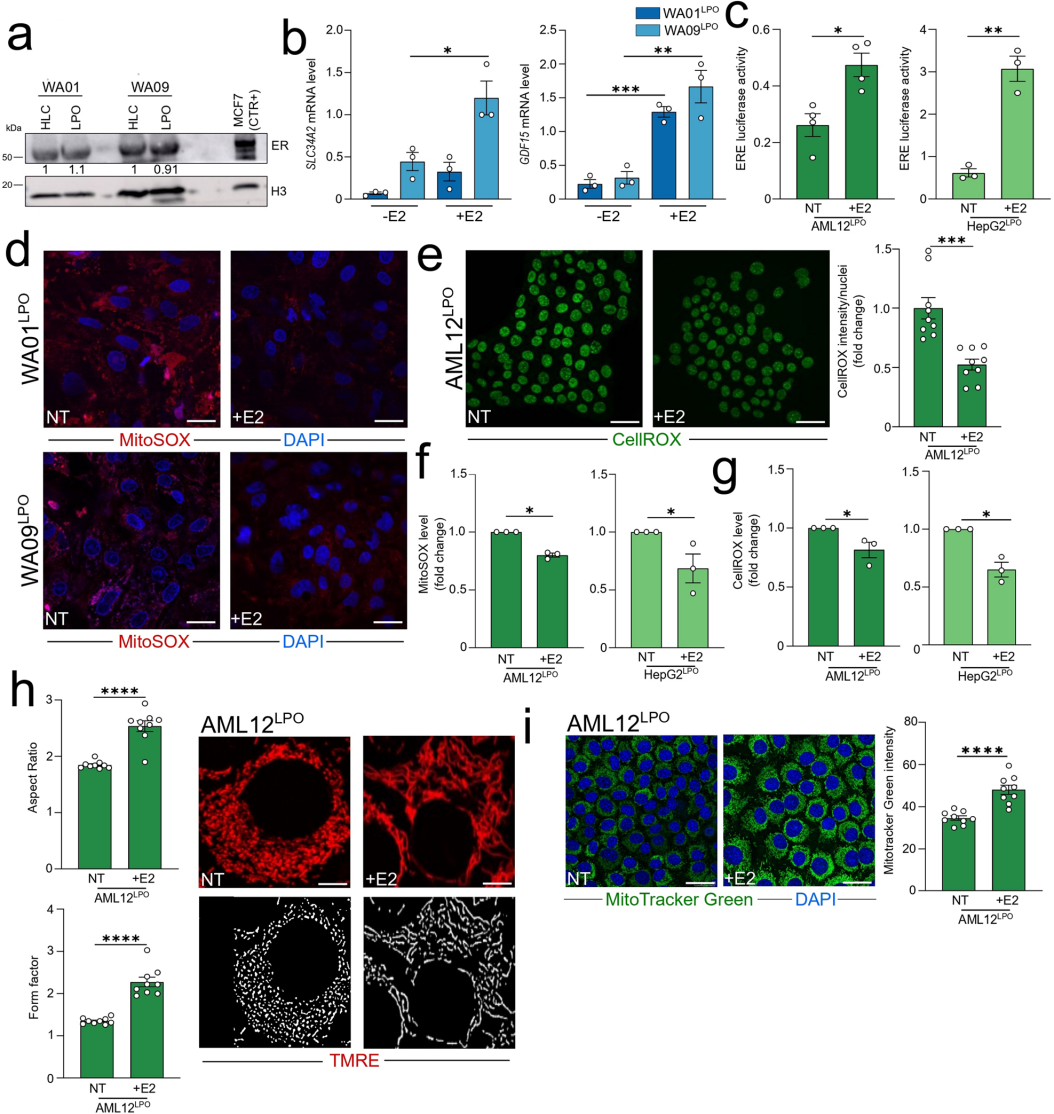
Estrogens reduced oxidative stress and altered mitochondrial morphology in LPO-treated hepatocytes. **a** Untreated and LPO-exposed WA01 and WA09 HLC were subjected to western blot analysis with the antibodies indicated. ER+ MCF7 breast cancer cell line is used as a positive control (CTR+) for estrogen receptor (ER) expression. The Histone H3 (H3) is used as a protein loading control normalizer. **b** LPO-treated WA01 and WA09 HLC were cultured with or without 1 nM of 17β-estradiol (E2) for 48 h and subjected to qRT-PCR using the assays described in the figure. The relative quantity is shown using ^∆^Ct. **c** LPO-treated AML12 and HepG2 cells were cultured with or without 50 nM of E2 for 48 h and subjected to ERE-luciferase reporter assay. **d** LPO-treated WA01 and WA09 HLC were cultured with or without 1 nM of E2 for 48 h and subjected to confocal analysis. Representative confocal images of MitoSOX-stained cells are shown (red: MitoSOX; blue: DAPI, nuclei. Scale bar, 25 µm). **e** LPO-treated AML12 cells were cultured with or without 50 nM of E2 for 48 h and subjected to confocal analysis. Representative images of CellROX-stained cells are shown (Green: CellROX. Scale bar, 25 µm). Quantification of CellROX intensity is reported. **f**, **g** Intracellular ROS levels were measured by CellROX and MitoSOX staining in LPO-treated AML12 and HepG2 cells cultured with or without 50 nM of E2 for 48 h. The fluorometric values of the NT and E2-treated AML12 and HepG2 cells have been used as reference in Fig. 6b. **h** LPO-treated AML12 cells were cultured with or without 50 nM of E2 for 48 h and subjected to TMRE staining and subsequent quantification of mitochondrial morphology parameters: aspect ratio and form factor (Red: TMRE. Scale bar, 25 µm). The confocal analysis of LPO-treated AML12 cells has been used as reference in Fig. 3d and has been processed in parallel with E2-treated cells. **i** Representative images of MitoTracker Green-stained LPO-treated AML12 cells, with or without 50 nM of E2 for 48 h, are shown (Green: MitoTracker Green; blue: DAPI, nuclei. Scale bar, 25 µm). Quantification of MitoTracker Green is reported. The confocal analysis of LPO-treated AML12 cells has been used as reference in Fig. 3e and has been processed in parallel with E2-treated cells. Data represent means ± SEM. (**b**—GDF15; **c**; **e**; **h**; **i**) Student’s *t*-test, (**b**—SLC34A2; **f**; **g**) Mann–Whitney, *p < 0.05, **p < 0.01, ***p < 0.001, ****p < 0.0001. **a**–**g** Each dot represents a biological replicate, **h**, **i** three biological replicates in technical triplicate.

In light of evidence suggesting a protective role of estrogens in MASLD onset and progression [30], we evaluated the effects of 17β-estradiol (E2) administration in LPO-treated cells. We first confirmed the expression of ER in both untreated and LPO-induced HLC (Fig. 4a). Consistent with this hypothesis, we observed that a 48-h E2 treatment activated transcription of established E2-dependent, liver-specific genes [31], such as growth differentiation factor 15 (GDF15) and/or solute carrier family 34 member 2 (SLC34A2), in LPO-treated HLC (Fig. 4b).

Due to the limited transfectability of hESC-derived HLC, we employed AML12 and HepG2 cells, which facilitate genetic manipulation and analysis. Using these cell lines, endogenous ER activity was monitored by a luciferase reporter assay in LPO condition following E2 administration (Fig. 4c), supporting the hypothesis that E2-induced transcriptional programming is mediated via ER. The transcriptional activation observed in response to E2 in the LPO models had significant functional implications: notably, a 48-h E2 treatment reduced mitochondrial ROS levels in WA01- and WA09-derived HLC (Fig. 4d). Additionally, the elevated total and mitochondrial ROS levels observed with LPO and MASH-inducing treatments in AML12 and HepG2 cells were similarly attenuated following E2 administration, as detected by MitoSOX and CellROX (Fig. 4e–g, S1b). E2 administration also increased the mitochondrial morphological indicators, form factor and aspect ratio (Fig. 4h), and mitochondrial mass (Fig. 4i). These analyses demonstrated that the fragmented mitochondrial morphology in LPO-treated cells was reversible with E2 treatment, potentially accounting for the observed reduction in ROS levels.

**Fig. 5:**
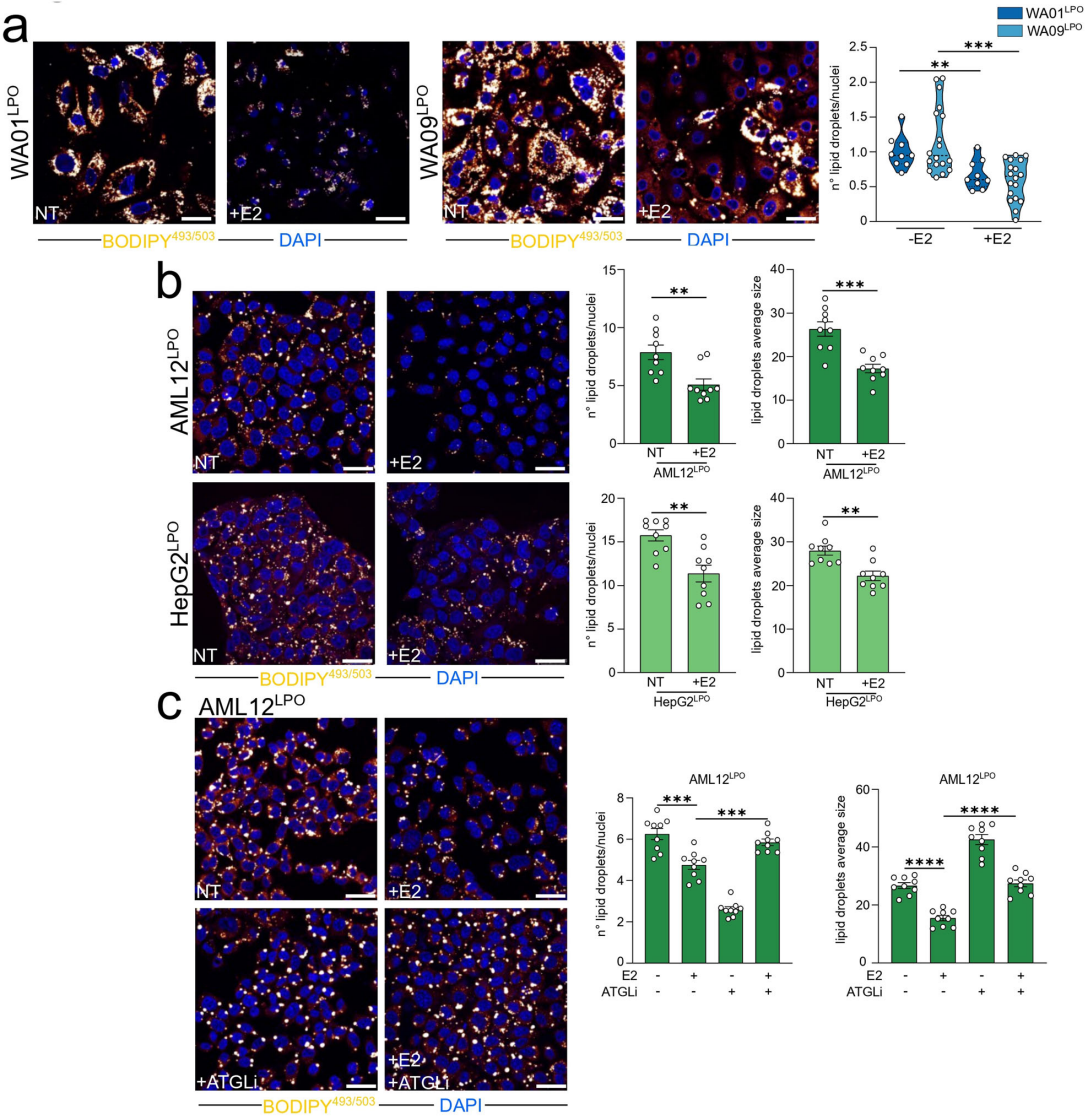
Estrogens reduced LD accumulation in LPO-treated hepatocytes. **a** LPO-treated WA01 and WA09 HLC were cultured with or without 1 nM of E2 for 48 h and subjected to confocal analysis. Representative confocal images of BODIPY^493/503^ stained cells are shown (orange/yellow: LD; blue: DAPI, nuclei. Scale bar, 25 µm). Quantification of BODIPY^493/503^ spots/cell is reported. **b** LPO-treated AML12 and HepG2 were cultured with or without 50 nM of E2 for 48 h and subjected to confocal analysis. Representative confocal images of BODIPY^493/503^ stained cells are shown (orange/yellow: LD; blue: DAPI, nuclei. Scale bar, 25 µm). Quantification of LD number/cell and their average size (arbitrary unit) is reported. The confocal analysis of LPO-treated AML12 cells and HepG2 cells have been used as reference in Fig. 2b and has been processed in parallel with E2-treated cells. **c** LPO-exposed AML12 cells were treated with 50 nM of E2 for 48 h and 20 µM of ATGL inhibitor (ATGLi) for 24 h. Representative confocal images of BODIPY^493/503^ stained cells are shown (orange/yellow: LD; blue: DAPI, nuclei. Scale bar, 25 µm). Quantification of LD number/cell and their average size (arbitrary unit) is reported. Data represent means ± SEM. **a**–**c** Student’s *t*-test, **p < 0.01, ***p < 0.001, ****p < 0.0001. **a**–**c** *n* ≥ 3 in three technical replicates.

The impact on redox balance of E2 administration was paralleled by alteration in the LD number and content of LPO-treated cells. Indeed, a 48-h E2 treatment reduced number and/or size of LD in WA01- and WA09-derived HLC (Fig. 5a), AML12 and HepG2 (Fig. 5b) cells that were exposed to LPO. The intracellular lipid content could be influenced by the ability to promote LD biogenesis and lipid mobilization from LD, a process largely dependent on adipose triglyceride lipase (ATGL) [32]. ATGL inhibition (ATGLi) did abrogate the effects of E2 in lowering the size and number of LD in LPO-exposed AML12 cells (Fig. 5c), thus suggesting that the observed E2 effects could be largely mediated by boosting lipid mobilization from internal depots.

Interestingly, both male- and female-derived HLC showed comparable responses to E2 administration, suggesting that the “gender-relevant phenotype” is independent of the HLC genetic background. Collectively, these results underscore the potential role of E2 in protecting against cell lipid accumulation, oxidative stress and mitochondrial dysfunction in our steatotic models.

### TRX2 inhibition reverted estrogen-mediated ROS buffering

**Fig. 6:**
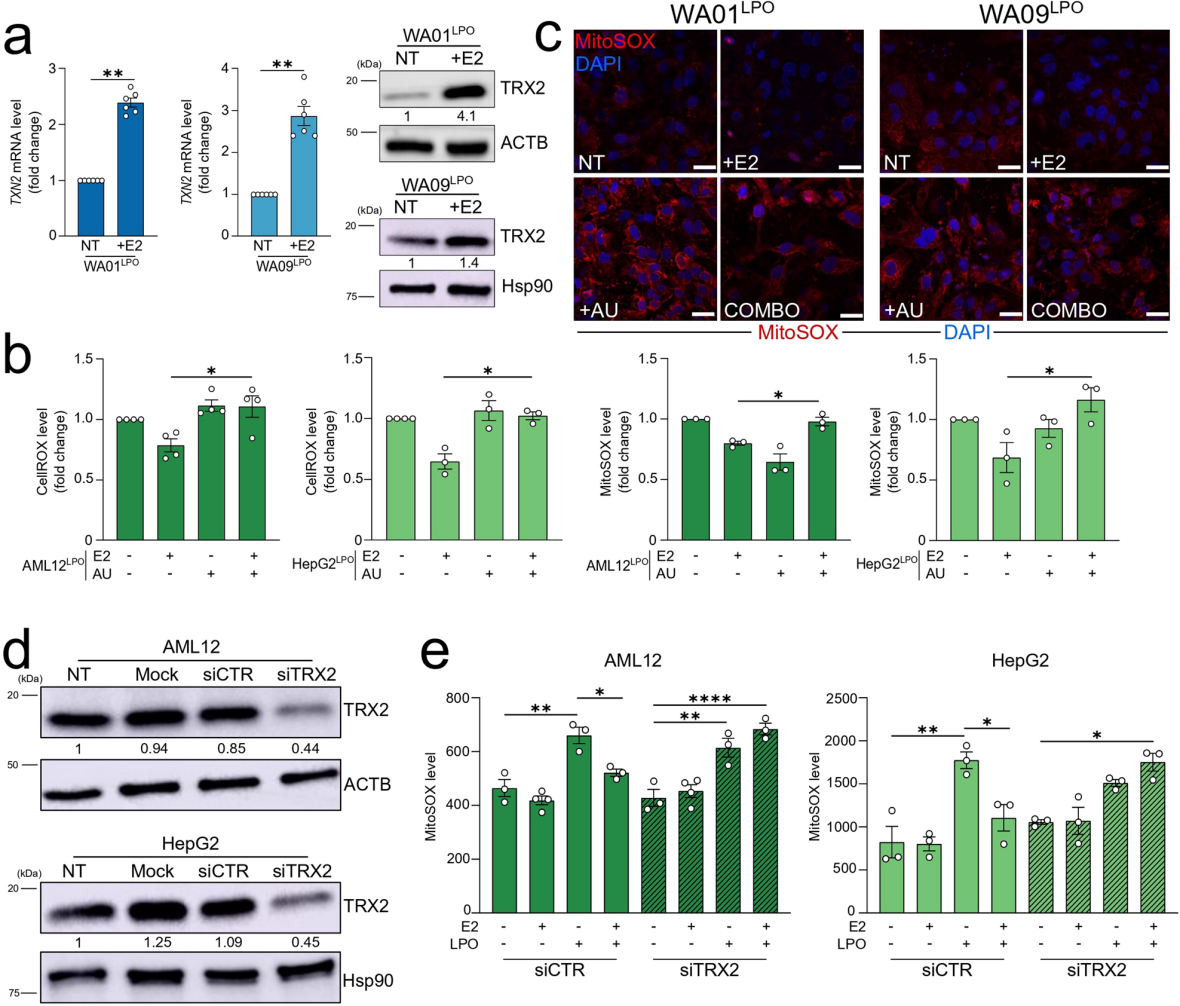
TRX2 inhibition reverted estrogen-mediated ROS buffering. **a** LPO-treated WA01 and WA09 HLC were cultured with or without 1 nM E2 for 48 h and subjected to qRT-PCR and western blotting analysis using the assays described in the figure. The Hsp90 and ACTB are used as protein-loading control normalizers. **b** ROS levels were measured with CellROX and MitoSOX probes in LPO-treated AML12 and HepG2 cells cultured with or without 50 nM of E2 and/or 250 nM of auranofin (AU) for 48 h. The fluorometric values of the NT and E2-treated AML12 and HepG2 cells are those reported in Fig. 4f, g and have been processed in parallel with AU-treated cells. **c** LPO-treated WA01 and WA09 cells were cultured with or without 1 nM of E2 and/or 500 nM of auranofin (AU) for 48 h and subjected to confocal analysis. Representative images of MitoSOX-stained cells are shown (Red: MitoSOX; blue: DAPI, nuclei. Scale bar, 25 µm). **d** Total protein lysates from AML12 and HepG2 cells transfected with 50 nmol/L of siTRX2 or siCTR for 72 h were subjected to western blot analysis, as indicated. The Hsp90 and ACTB are used as protein-loading control normalizers. **e** Mitochondrial ROS levels were measured using MitoSOX probe in TRX2-silenced AML12 and HepG2 cells treated with or without LPO and/or 50 nM of E2 for 48 h. Data represent means ± SEM. **a** Mann–Whitney, (**b**; e-HepG2) Kruskal–Wallis, Dunnett-corrected, (**e**—AML12) One-way ANOVA, Tukey-corrected, *p < 0.05, **p < 0.01, ***p < 0.001, ****p < 0.0001. Each dot represents a biological replicate.

To elucidate the mechanism underlying the protective effects of E2, we investigated the involvement of antioxidant systems previously reported to be regulated by E2. Surprisingly, NRF2, a well-known ER-dependent gene [33, 34], did not respond to E2 treatment in terms of expression or activation, as indicated by unchanged levels of the downstream target gene NQO1 in HLC (Fig. S2). Consequently, we focused on TRX2, a component of the mitochondrial thioredoxin system known to be regulated by E2 [16], as a possible mediator of the E2-driven ROS buffering observed in LPO-treated models. Based on the presence of an estrogen-responsive element in the *TXN2* promoter (Harmonizome 3.0, using JASPAR Predicted Transcription Factor Targets dataset for *ESR1*: https://maayanlab.cloud/Harmonizome/gene_set/ESR1/JASPAR+Predicted+Transcription+Factor+Targets), we hypothesized that TRX2 is directly regulated by E2. Our time-course analysis confirmed that E2 increased *TXN2* mRNA levels as early as 2–4 h post-treatment in AML12 cells, while protein levels were elevated up to 48 h in AML12 and HepG2 cell lines (Fig. S3a–c). Consistent with this hypothesis, E2 treatment resulted in elevated *TXN2* mRNA and TRX2 protein levels in LPO-exposed WA01 and WA09 HLC (Fig. 6a).

To assess the role of the TRX2 system in the protective effects of E2, we first used auranofin, a pharmacological agent approved for rheumatoid arthritis treatment, which is known to disrupt the TRX2 antioxidant pathway [35]. In LPO-exposed cellular models, auranofin treatment reversed the antioxidant effects of E2, as evidenced by increased mitochondrial and total ROS levels in both immortalized and HLC models (Fig. 6b, c). It is important to note, however, that auranofin administration in LPO-exposed cells independently induced oxidative stress, suggesting that auranofin may exert a pro-oxidant effect, potentially through direct interaction with the TRX2 system or by influencing other redox regulatory mechanisms. Therefore, to exclude any confounding effects caused by this pharmacological inhibition, we adopted a targeted siRNA approach to selectively inhibit TRX2 in immortalized cells (Fig. 6d). Notably, TRX2 silencing reversed the antioxidant effects of E2 in both LPO-exposed AML12 and HepG2 cell lines (Fig. 6e).

### TXN2 expression profile in a cohort of female MASLD patients

To assess the clinical relevance of our findings, we investigated whether *TXN2* expression correlates with different stages of MASLD progression and could be associated with disease onset. Subudhi *et al*. conducted a comprehensive analysis of approximately 800 genes in liver tissue samples from 318 donors, classified into four histological categories: normal liver histology (Normal), steatosis only (Steatosis), MASH without fibrosis (early MASH), and MASH with fibrosis stages 1–4 (late MASH) [36]. For our analysis, we extracted *TXN2* expression data specifically from the 243 female liver samples and categorized the specimens into pre- and postmenopausal groups, using age 50 as the threshold.

**Fig. 7:**
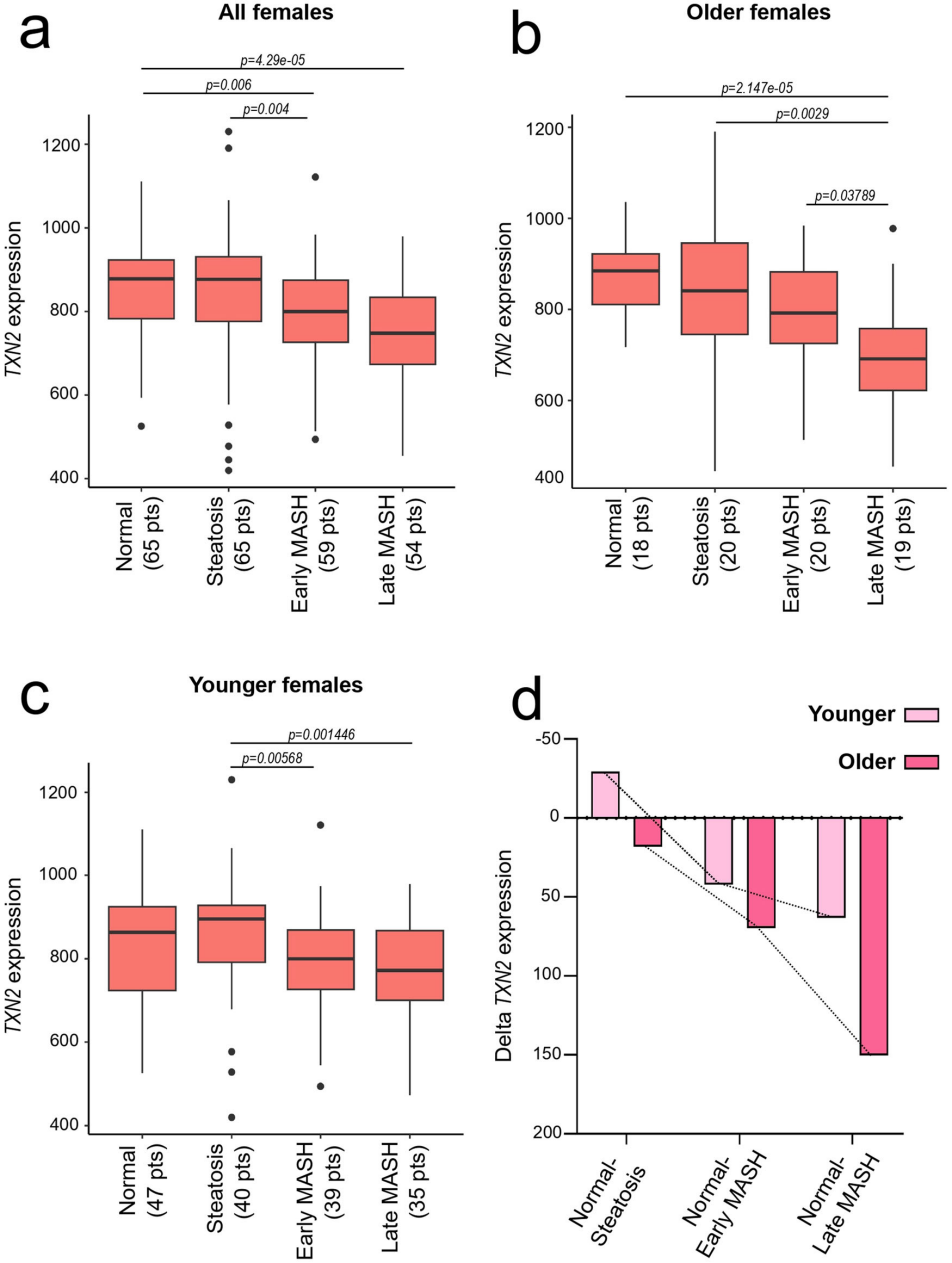
TXN2 expression profile in a cohort of female MASLD patients. Gene expression of *TXN2* was analyzed with GEO2R and *R*-program in a cohort of normal or MASLD-bearing patients (GSE163211) divided by age. *TXN2* expression levels **a** in the whole female population, **b** in the postmenopausal (Age ≥ 50), and **c** in premenopausal women (Age < 50). **d** Differences in *TXN2* expression between postmenopausal and premenopausal women compared to normal tissue (Normal-Steatosis, Normal-early MASH, and Normal-late MASH stage). Wilcox test.

While *TXN2* expression levels in postmenopausal healthy donors were comparable to or slightly higher than those in premenopausal healthy donors (normal: mean premenopausal = 831.45, mean postmenopausal = 878.57), *TXN2* expression was notably higher in premenopausal specimens at all three stages of MASLD progression compared to their postmenopausal counterparts (Steatosis: mean premenopausal = 860.93, mean postmenopausal = 834.93; Early MASH: mean premenopausal = 789.21, mean postmenopausal = 783.47; Late MASH: mean premenopausal = 768.25, mean postmenopausal = 702.79; Fig. 7a–c). Notably, the decline in *TXN2* expression among postmenopausal patients became more pronounced as MASLD advanced (Fig. 7d), underscoring the potential clinical importance of TRX2 monitoring in MASLD progression.

Given the critical role of ROS formation and regulation in MASLD, it is plausible that the reduced presence of estrogens in postmenopausal women—and the subsequent decrease in estrogen-dependent, TRX2-mediated ROS scavenging—contributes to the more marked decline in *TXN2* expression observed in this cohort as the disease progresses.

## Discussion

MASLD is a global health problem, with its prevalence on the rise, that is characterized by the association of liver steatosis to additional comorbidities including obesity, diabetes, and cardiovascular and renal diseases [1].

The incidence of MASLD displays a notable disparity between the sexes, with men exhibiting a higher prevalence compared to premenopausal women [37]. This observation underscores the pivotal role of sex hormones, particularly estrogens, in MASLD pathogenesis. Indeed, the decline in estrogen levels in postmenopausal women contributes to an increased susceptibility to MASLD, highlighting estrogens’ significance in the context of liver health [38]. Estrogens are known to exert protective effects against hepatic lipid accumulation through various mechanisms, including the modulation of lipid metabolism, insulin sensitivity, and inflammation [39]. A recent study by Meda and colleagues found that the loss of estrogens in women with obesity induces a shift in liver gene expression toward a male-like profile, driven by ER. This shift is linked to the onset of MASLD and suggests that sex-specific approaches, particularly for postmenopausal women, could be effective in preserving liver health and mitigating MASLD progression [40]. The current study aimed to expand further this area of investigation, increasing the mechanistic insights involved in the estrogen-mediated protective role.

Since both genomic and environmental factors contribute to the onset and progression of MASLD, it is difficult to study the molecular mechanisms underlying the disease and, consequently, to find effective therapeutic approaches. A further aspect complicating the study of MASLD is the lack of in vitro and in vivo experimental models capable of recapitulating the complexity of the liver tissue. Currently, in vivo murine models serve as the primary research tool for MASLD because they have demonstrated a higher likelihood of extrapolating findings from studies on the disease’s pathophysiology in humans and a more accurate assessment of possible therapeutic targets [41]. On the other hand, utilizing in vitro models allows a more precise manipulation and observation of specific pathways and better control over experimental variables, essential for elucidating the underlying mechanisms involved in liver diseases.

In this study, we complemented immortalized liver cell lines with HLC obtained from hESC that were subjected to LPO administration. LPO generated hepatic steatosis and increased ROS levels in all the cell models employed. Such an approach allowed the establishment of a robust platform for studying mechanistic determinants involved in MASLD onset and progression, including the role of estrogens.

Besides its role in reproduction and sexual development, estrogens can influence the development of several complex pathologies, including metabolic and liver disease [22]. Indeed, the prevalence of metabolic syndrome rises with menopause, most likely as a secondary effect of the metabolic reprogramming from central fat redistribution induced by reduced levels of estrogens [31], since it is known that estrogens regulate the activity of a series of enzymes involved in de novo synthesis and oxidation of fatty acids [42]. Indeed, also in experimental conditions, estrogens reduce the susceptibility to steatosis development in liver cells of female mice fed a high-fat diet (HFD) subjected to ovariectomy [24]. Furthermore, independent of genetic background, ER deletion in Western-type diet-fed mice of both sexes resulted in impaired high-density lipoprotein (HDL) internalization by isolated hepatocytes. Interestingly, this effect was observed despite elevated serum cholesterol and HDL levels specifically in female mice [43].

**Fig. 8:**
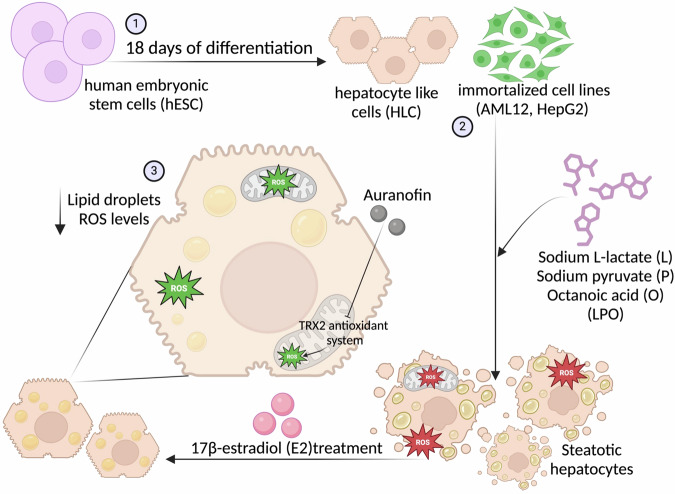
Graphical abstract of the study. (1) Human embryonic stem cells (hESC) were differentiated into hepatocyte-like cells (HLC) using an 18-day differentiation protocol; (2) HLC and hepatic immortalized cell lines (AML12, HepG2) were then treated with sodium L lactate (L), sodium pyruvate (P) and octanoic acid (O), a protocol of induction called LPO. LPO administration induces distinct steatotic traits, such as the increase of lipid droplets and reactive oxygen species (ROS) levels. (3) Treatment with 17-β estradiol (E2) results in a reduction of the distinctive traits of MASLD through the activation of the mitochondrial antioxidant mechanism of thioredoxin 2 (TRX2), which is inhibited by the administration of auranofin.

While dysfunction in lipid metabolism should be considered the basis of MASLD onset, its progression requires multiple additional hits, including oxidative stress [44]. It has been reported that estrogens can reduce oxidative stress and cell damage in cardiovascular diseases [45], where the incidence, like MASLD, is much higher in men and postmenopausal women than in fertile women. Specifically, it has been reported that E2 reduces ROS production and protects against oxidative stress by increasing the activation of the NRF2-mediated antioxidant response [46]. This led us to hypothesize that such a mechanism could also be implicated in cyto-protection during MASLD. We, therefore, verified if E2 administration could lower ROS levels in our in vitro experimental models, preventing or postponing the disease’s progression. While we noted that E2 played a protective role in reducing the enhanced levels of ROS and lipid accumulation associated with LPO-induced MASLD independently from genetic background (Fig. 8), we did not observe a clear involvement of NRF2 activation (Fig. S2). Nonetheless, we identified TRX2 as implicated in the protective mechanism dependent on E2 in our experimental setups. TRX2, localized within mitochondria, serves as a crucial antioxidant defense system, scavenging ROS and maintaining mitochondrial redox balance (Fig. 8). Dysregulation of TRX2 could exacerbate mitochondrial dysfunction, perpetuating oxidative stress and contributing to MASLD progression. Indeed, strategies aimed at enhancing TRX2 activity or expression hold the potential to mitigate oxidative stress and subsequent liver injury in MASLD [47].

An aspect not investigated in our study pertained to the potential interaction between E2, ER, and TRX2, particularly behind the E2-dependent transcriptional regulation of TRX2. The interplay between TRX2 and estrogens can occur at multiple levels: as shown in our study, TRX2 appears to be an E2-dependent gene. However, estrogens can influence ROS levels regulating mitochondrial biogenesis and subsequent function. Since TRX2 is primarily located in the mitochondria, E2 effects on mitochondrial function may also impact TRX2 activity. These effects can be reverted by the administration of the TRX2 inhibitor, namely auranofin, which resulted in an impairment of the antioxidant effects of estrogens in our MASLD models. Indeed, auranofin has been shown to induce oxidative damage and modifications of cellular redox status, resulting in the overproduction of ROS and apoptosis of gastric cancer cells [48]. Nonetheless, treatment with auranofin decreased palmitic acid-induced steatosis in HepG2 and lipogenesis and fibrosis in western diet-induced MASLD model mice [49].

Therefore, estrogens, through their antioxidant properties [50], can modulate redox signaling pathways, hence shaping the overall antioxidant cellular response: TRX2 may play a role and exert compensative antioxidant function, and therefore, the role we have described in our in vitro study has to be related to the MASLD pathogenesis. Further studies are needed to address the specific mechanisms underlying TRX2 and ER interaction and potential therapeutic implications, including also the importance of stromal and immune cells in this crosstalk, which will certainly participate in the progression of MASLD disease.

In conclusion, understanding the gender-specific disparities, the influence of estrogens, the significance of redox balance, and the role of the TRX2 system in MASLD onset and progression provides valuable insights into the pathophysiology of this complex disorder. Targeted therapeutic interventions aimed at restoring hormonal balance, preserving redox homeostasis, and enhancing mitochondrial function hold promise in mitigating MASLD burden and improving clinical outcomes. Collaborative efforts integrating basic research and clinical translation are imperative in advancing our understanding and management of MASLD in both sexes.

## Supplementary information

Supplementary Figures

Original uncropped blots

## Data Availability

Data will be made available from the corresponding author upon request.
