# Supplementary material for: Estrogen-dependent activation of TRX2 reverses oxidative stress and metabolic dysfunction associated with steatotic disease
Source: Cell Death Dis. 2025 Jan 31;16(1):57. doi: 10.1038/s41419-025-07331-7 (PMC11785963; doi:10.1038/s41419-025-07331-7)
Supplement: Supplementary file 1 — Supplementary Figures [file 41419_2025_7331_MOESM1_ESM.pdf]

SUPPLEMENTARY DATA

Estrogen-dependent activation of TRX2 reverses oxidative stress and metabolic dysfunction associated with steatotic disease

Alfredo Smiriglia, Nicla Lorito, Marina Bacci, Angela Subbiani, Francesca Bonechi, Giuseppina Comito, Marta Anna Kowalik, Andrea Perra and Andrea Morandi.

Corresponding author: andrea.morandi@unifi.it

The Supplementary Data contains:

Supplementary Figures 1-3

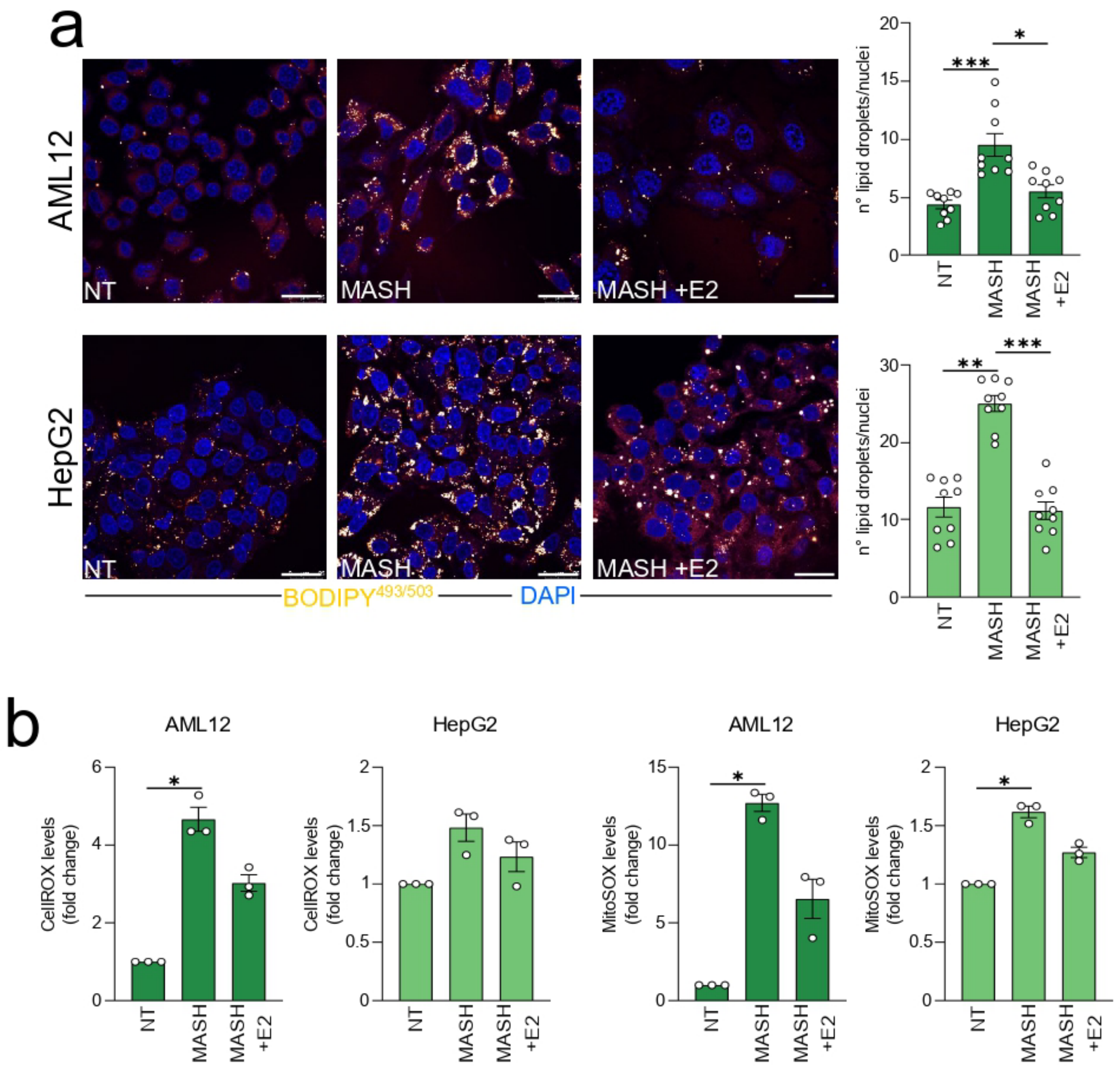

Supplementary Figure 1

(**a**) Untreated and MASH-induced AML12 and HepG2 cells were cultured with or without 50 nM of E2 for 48 hours and subjected to confocal analysis using $\text{BODIPY}^{493/503}$. Representative confocal images of $\text{BODIPY}^{493/503}$ stained cells are shown (orange/yellow: LD; blue: DAPI, nuclei. Scale bar, 25 μm). Quantification of $\text{BODIPY}^{493/503}$ spots/cell is reported. (**b**) ROS levels were measured with CellROX and MitoSOX probes in MASH-induced AML12 and HepG2 cells with E2-treatment. Data represent means ± SEM. Kruskal-Wallis, Dunnett-corrected, *p < 0.05, **p < 0.01, ***p < 0.001. (a) three biological replicates in technical triplicate, (b) Each dot represents a biological replicate.

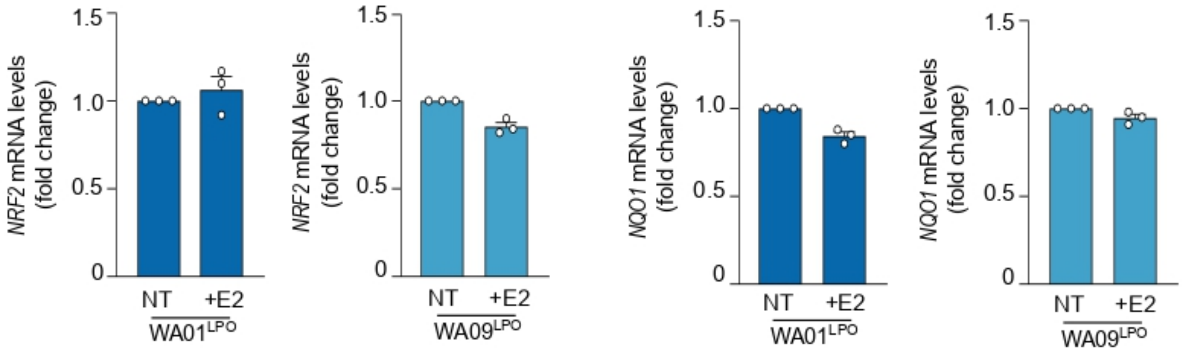

Supplementary Figure 2

LPO-treated WA01 and WA09 HLC were cultured with or without 1nM E2 for 48 hours and subjected to qRT-PCR analysis using the assays described in the Figure. Mann-Whitney revealed no statistically significant differences. Each dot represents a biological replicate.

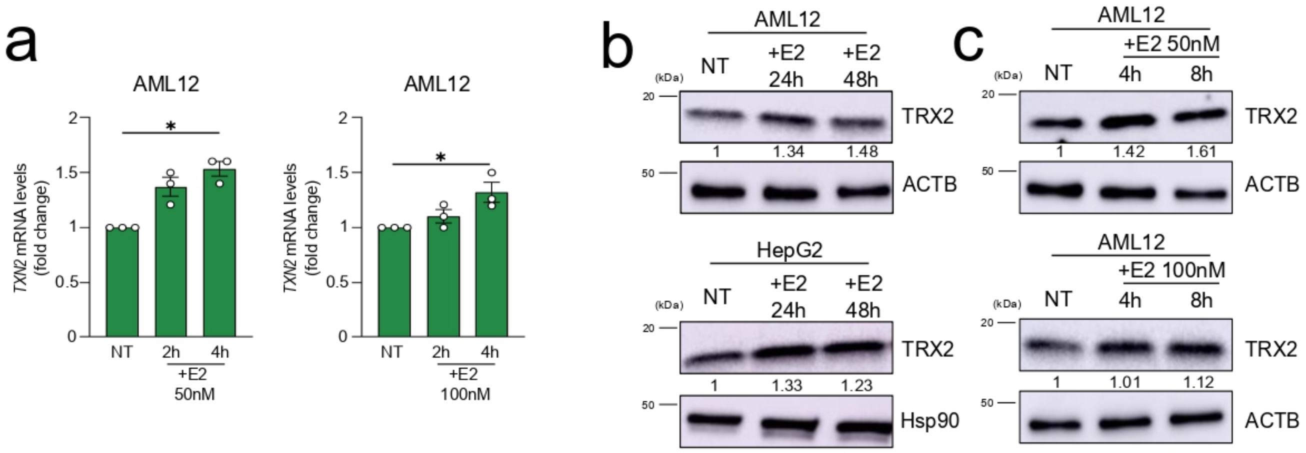

Supplementary Figure 3

(**a**) AML12 cell line was subjected to qRT-PCR analysis. (**b,c**) Total protein lysates from AML12 cell line were subjected to western blot analysis with the antibodies indicated. The ACTB is used as a protein loading control normalizer. The relative quantity normalized on untreated is shown using $^{\Delta\Delta}$Ct. Data represent means ± SEM. Kruskal-Wallis, Dunnett-corrected, *p < 0.05. Each dot represents a biological replicate.
