# Supplementary material for: Estrogen-dependent activation of TRX2 reverses oxidative stress and metabolic dysfunction associated with steatotic disease
Source: Cell Death Dis. 2025 Jan 31;16(1):57. doi: 10.1038/s41419-025-07331-7 (PMC11785963; doi:10.1038/s41419-025-07331-7)

Supplemental Material – Smiriglia et al.

ORIGINAL DATA

Full and Uncropped original western blots displayed in Figure 1c

HNF4α

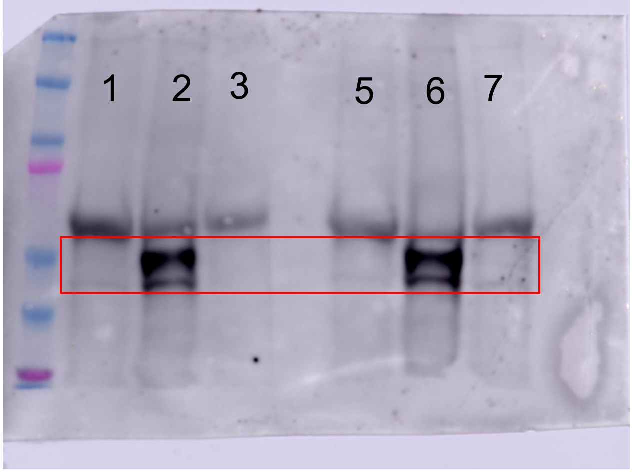

Line 1: WA01 hESC
Line 2: WA01 HLC
Line 3: WA01 LPO
Line 5: WA09 hESC
Line 6: WA09 HLC
Line 7: WA09 LPO

E-CAD

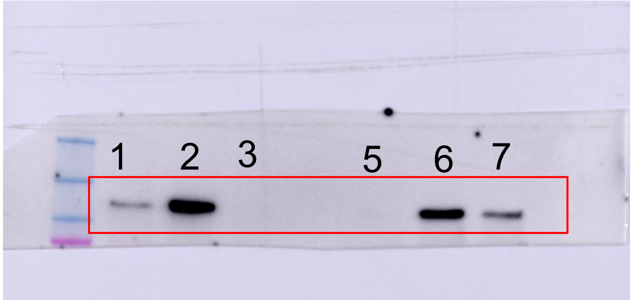

HISTONE H3

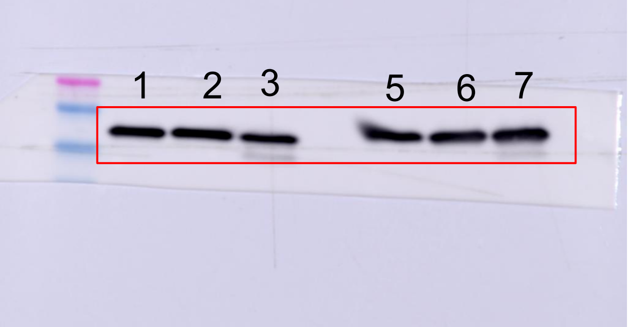

Full and Uncropped original western blots displayed in Figure 4a

ER

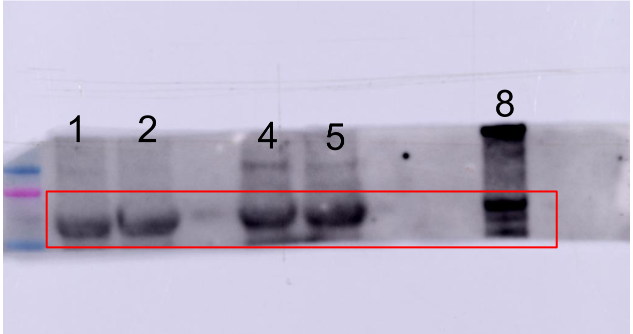

Line 1: WA01 HLC
Line 2: WA01 LPO
Line 4: WA09 HLC
Line 5: WA09 LPO
Line 8: MCF7 (CTR+)

HISTONE H3

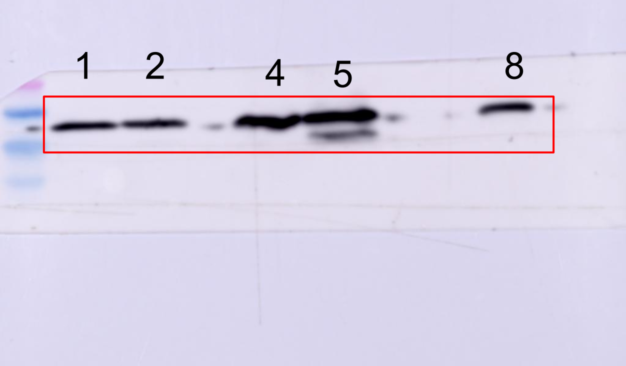

Full and Uncropped original western blots displayed in Figure 6a

TRX2 (WA01)

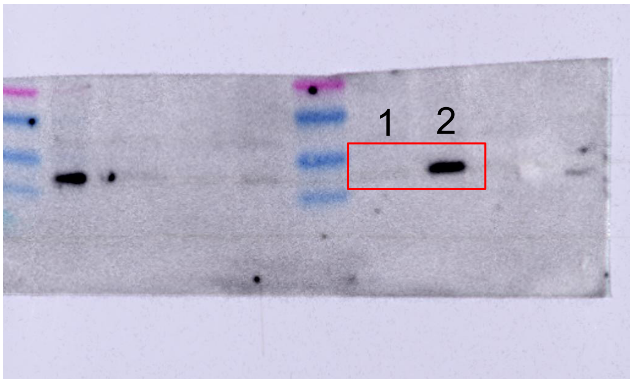

Line 1: WA01 LPO -E
Line 2: WA01 LPO +E

ACTB (WA01)

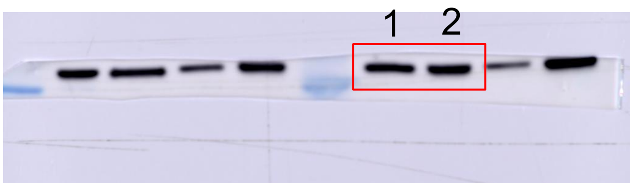

TRX2 (WA09)

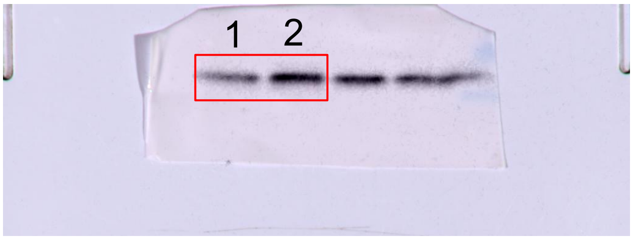

Line 1: WA09 LPO -E
Line 2: WA09 LPO +E

Hsp90 (WA09)

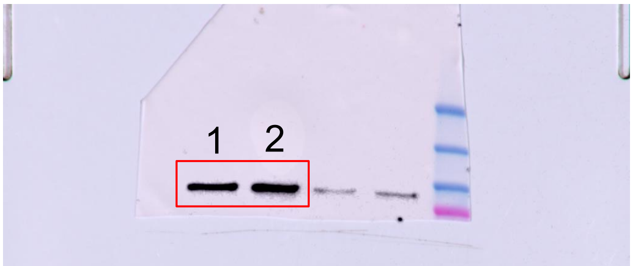

Full and Uncropped original western blots displayed in Figure 6d

TRX2 (AML12)

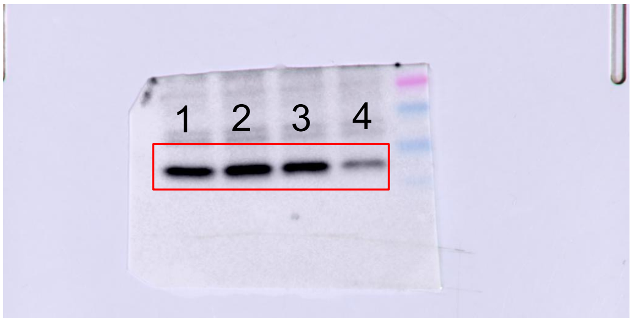

ACTB (AML12)

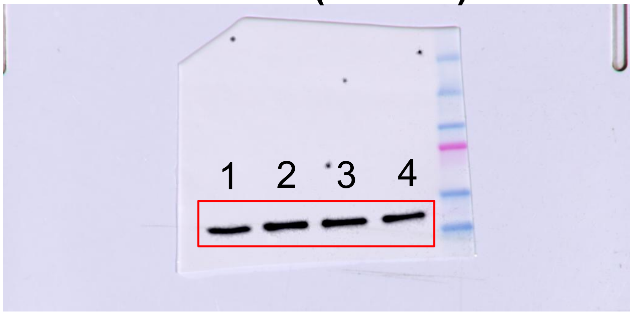

Line 1: AML12 NT
Line 2: AML12 MOCK
Line 3: AML12 siCTR
Line 4: AML12 siTRX2

TRX2 (HepG2)

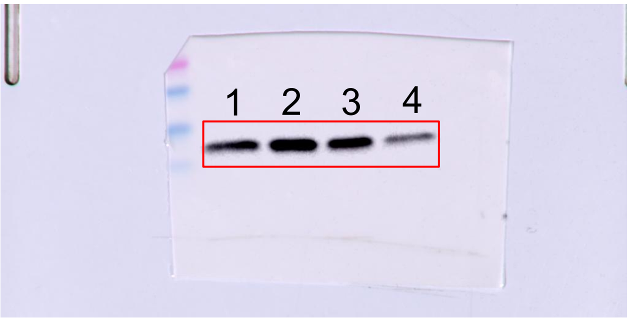

Hsp90 (HepG2)

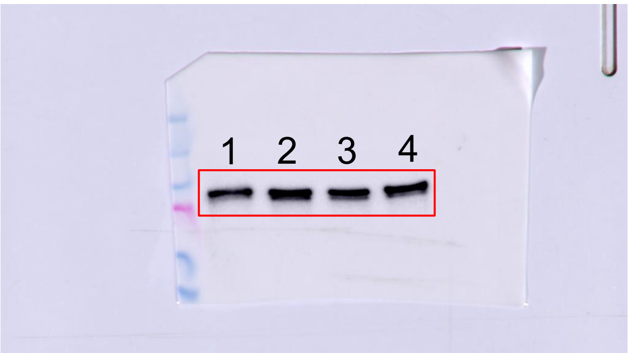

Line 1: HepG2 NT
Line 2: HepG2 MOCK
Line 3: HepG2 siCTR
Line 4: HepG2 siTRX2

Full and Uncropped original western blots displayed in Figure S3b

TRX2 (AML12)

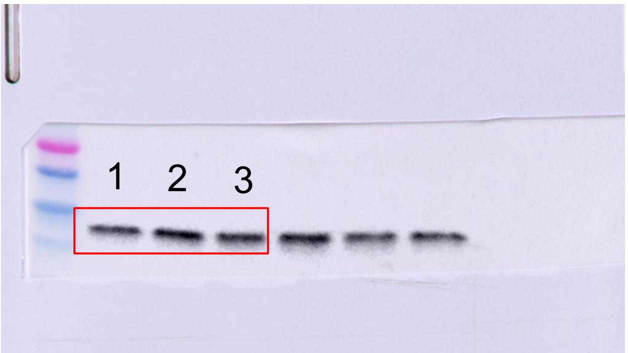

ACTB (AML12)

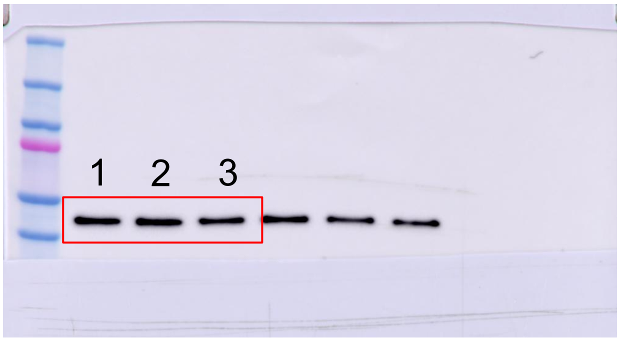

Line 1: AML12 NT
Line 2: AML12 +E2 24h
Line 3: AML12 +E2 48h

TRX2 (HepG2)

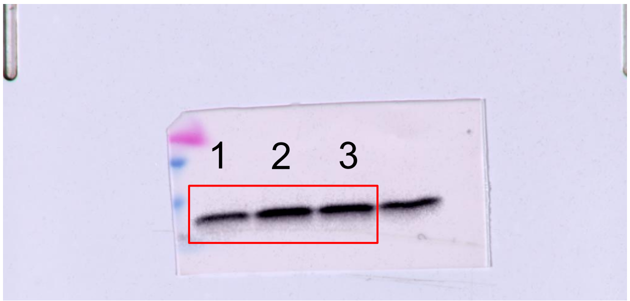

Hsp90 (HepG2)

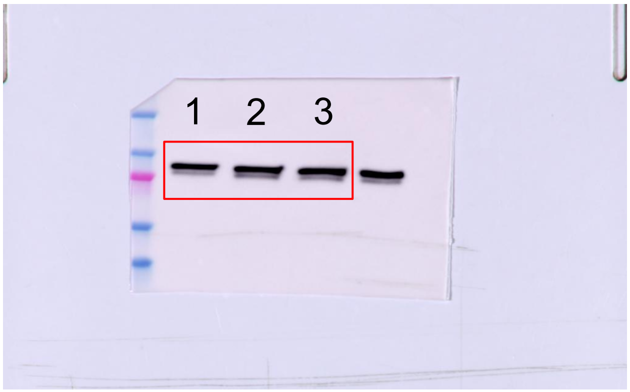

Line 1: HepG2 NT
Line 2: HepG2 +E2 24h
Line 3: HepG2 +E2 48h

Full and Uncropped original western blots displayed in Figure S3c

TRX2 (AML12)

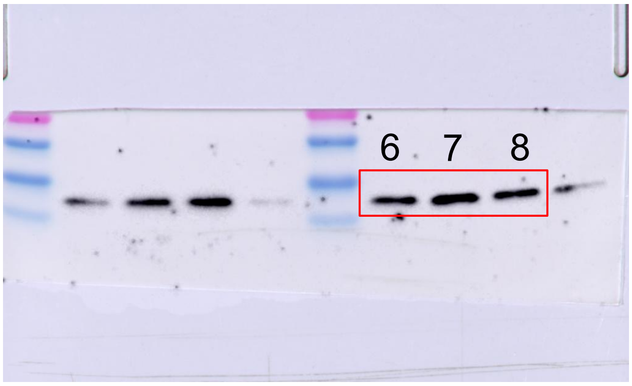

TRX2 (AML12)

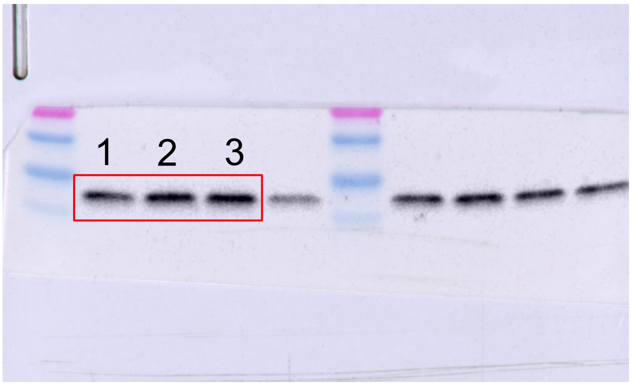

Line 1: AML12 NT
Line 2: AML12 +E2 100nM 4h
Line 3: AML12 +E2 100nM 8h

Line 6: AML12 NT
Line 7: AML12 +E2 50nM 4h
Line 8: AML12 +E2 50nM 8h

ACTB (AML12)

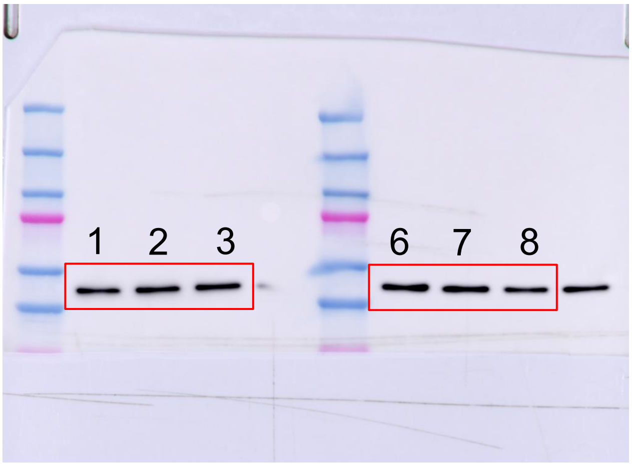

Supplement: Supplementary file 2 — Original uncropped blots [file 41419_2025_7331_MOESM2_ESM.pdf]
